# Putting prospection into practice: Methodological considerations in the use of episodic future thinking to reduce delay discounting and maladaptive health behaviors

**DOI:** 10.3389/fpubh.2022.1020171

**Published:** 2022-11-03

**Authors:** Jeremiah Michael Brown, Jeffrey Scott Stein

**Affiliations:** ^1^Fralin Biomedical Research Institute, Virginia Tech Carilion, Roanoke, VA, United States; ^2^Department of Human Nutrition, Foods, and Exercise, College of Agriculture and Life Sciences, Virginia Tech, Blacksburg, VA, United States

**Keywords:** episodic future thinking, delay discounting, health behavior change, methods, narrative review

## Abstract

In recent years, episodic future thinking (EFT) has emerged as a promising behavioral intervention to reduce delay discounting or maladaptive health behaviors; however, considerable methodological heterogeneity in methods for eliciting engagement in EFT has been observed in prior research. In this narrative review, we briefly describe methods for generating EFT cues, the content of EFT cues, common control conditions for experiments utilizing EFT, and considerations for cue delivery and implementation. Where possible, we make suggestions for current best practices in each category while identifying gaps in knowledge and potential areas of future research. Finally, we conclude by using the NIH Stage model to better frame the current state of the literature on EFT and propose gaps to be addressed if EFT is to be both an efficacious and effective behavioral intervention.

## Introduction

Episodic future thinking (EFT) is the act of imagining personal future events and experiences in great detail (1). EFT is distinct from future thought focusing on generalized knowledge of upcoming events, called semantic future thinking (e.g., imagining the experience of traveling to France to watch the 2023 Rugby World Cup vs. knowing that the 2023 Rugby World Cup will be held in France), and typically involves events with a discrete beginning and end. In recent years, EFT has been applied as an agent of behavior change to a growing number of health behaviors and populations to reduce delay discounting [i.e., the tendency of a reinforcer to decrease in value as the time to receive it increases; for overview, see (2)]. Delay discounting, a measure of how individuals value future consequences, is cross-sectionally and longitudinally associated with a range of maladaptive health behavior and lifestyle-related disease (3). For example, higher rates of delay discounting are associated with greater energy consumption, more sedentary activity, and higher body mass index [BMI; (4, 5)], as well as cigarette smoking (6, 7) and other substance use (8, 9).

**Figure 1:**
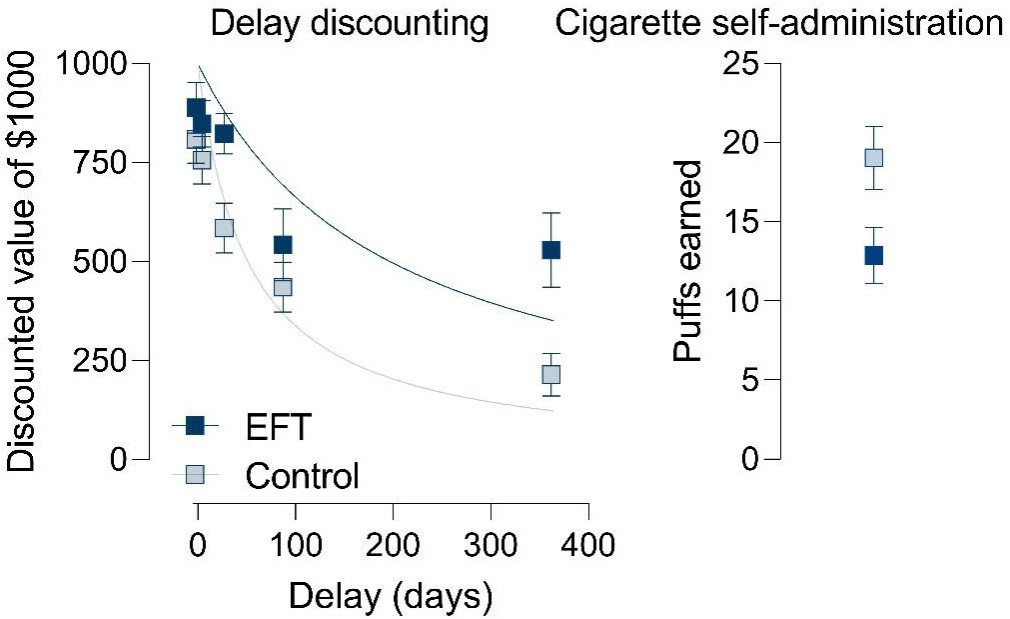
Example data, replotted from Stein et al. (10), showing effects of an episodic future thinking (EFT) intervention on delay discounting **(left)** and the number of cigarette puffs earned during a self-administration task **(right)** in cigarette smokers.

When applied in order to reduce delay discounting, several studies have shown that EFT also influences outcomes relevant to these health behaviors (see Figure 1 for an example). Specifically, acute delivery of EFT in the laboratory has been shown to reduce caloric consumption of highly palatable foods during an *ad-libitum* eating task in adults and children with overweight/obesity (11, 12) and in young adults with high BMI (13). Likewise, this form of EFT has reduced purchasing of high calorie and low nutrient foods in an online grocery shopping task (14). Similarly, acute delivery of EFT online reduces hypothetical demand for obesogenic fast foods in adults at risk for type 2 diabetes, even when challenged by simulations of economic scarcity (15). Using an alternative procedure to evoke episodic prospection, Kuo et al. (16) exposed weight-loss seeking undergraduates to weight-reduced avatars in a virtual fitting room, and observed both reduced rates of delay discounting and reduced ice cream consumption during a taste test compared to participants shown present-weight avatars. Applied to substance use, acute lab-based or online engagement in EFT has been shown to decrease self-administration of cigarettes and hypothetical cigarette demand in current smokers (10, 17, 18), and hypothetical alcohol demand in individuals with alcohol use disorder (19–21).

In natural settings, acute engagement in EFT has been used to reduce total calorie consumption and calories from fat in women with overweight/obesity who generated EFT cues in the lab, then listened to recordings of their cues while eating dinner in a food court the next day (22). Likewise, three EFT exposures (one in the lab, two at home) have been demonstrated to reduce purchasing of calories, grams of fat, and milligrams of sodium during weekly food shopping in mothers with overweight or obesity who engage in grocery shopping for their household (23).

These promising laboratory and online findings have led to the development of clinical applications, in which participants engage in EFT repeatedly in the natural environment *via* smartphone or other technology. Preliminary work in this area suggests that this form of remotely delivered EFT facilitates weight loss in adults with overweight and obesity (24) and reduces alcohol consumption in adults with alcohol use disorder (25). Likewise, periodic engagement in EFT may increase medication adherence in adults with type 2 diabetes or prediabetes (26).

In studies examining the effects of EFT on delay discounting and health behavior, participants typically generate text and/or audio descriptions of personally relevant future events; these event descriptions are then used as cues to prompt EFT during laboratory-based decision-making tasks [e.g., (27, 28)] or in the natural environment [e.g., (23, 25)]. While experiments involving EFT and delay discounting vary in their specific procedures, there are typically some commonalities. Generally, participants first generate EFT (or control) cues using interview-guided or survey-guided methods (discussed further in Section Cue generation methods). Following cue generation, participants may be instructed to vividly imagine or listen to audio recordings of their EFT or control cues during the delay discounting task (i.e., cued delay discounting; (29)). Alternatively, there may be no specific instructions to engage in EFT during the delay discounting task [i.e., uncued delay discounting; (30)]. For example, a cued delay discounting task may include the following instructions during a choice trial: Which would you prefer when you imagine: “In 10 years, I am visiting with relatives at a family reunion…”, while other studies may only present a shortened form of the full cue. Typically, delay discounting (or other behavioral) tasks occur immediately after cue generation, although this may depend on the specific research questions under investigation.

The ability of EFT to reduce delay discounting or excessive consumption of reinforcers could be the result of a change in the temporal window in which participants integrate the value of immediate vs. future rewards, allowing for a greater consideration of the utility of future rewards (31, 32). This hypothesized mechanism of action has been more formally theorized in Reinforcer Pathology theory (33–35). Described briefly, Reinforcer Pathology theory postulates that individuals who heavily discount the future may have difficulty integrating the value of delayed reinforcers (e.g., sustained good health and positive social interactions) and future negative outcomes (e.g., poor health outcomes and financial strain) into present decision making [see (36)]. Indeed, excessive delay discounting may be a trans-disease process underlying many maladaptive behaviors, such as drug usage and disordered gambling (3). Therefore, processes such as EFT that effectively decrease delay discounting and broaden the temporal window—allowing for a more complete synthesis of valuation between present and future consequences—may be targeting a mechanism central to the etiology of multiple behavioral disorders.

Additionally, recent work has examined the potential for demand characteristics to influence participant behavior during EFT-cued delay discounting tasks (37). Demand characteristics refer to a participant's ability to guess the hypothesis of the researcher which, in turn, may influence the participant's behavior in various ways. One common example of particular concern in EFT studies is the “good-subject effect” (38), in which participants deduce the experimental hypothesis and advertently or inadvertently adjust their behavior in accordance with this hypothesis. Previous online studies have demonstrated that demand characteristics are unlikely to account for the effects of EFT on delay discounting and health behavior (18, 30), although it is unknown if these findings would generalize to EFT's effects in the laboratory or clinical environments.

Indeed, the mechanisms underlying the effect of EFT on delay discounting and health behaviors are not fully understood. Moreover, little research has addressed how long these changes persist in the natural environment, and what participant characteristics (if any) may moderate the efficacy of EFT in specific populations. In order for EFT to have the ability to make a meaningful clinical impact, more empirical observations of EFT and behavior change designed to elucidate such moderating conditions are needed.

Such investigations may also aid in interpretation of null or contradictory findings in the literature. Specifically, despite the promising findings on EFT reviewed above, Bickel et al. (31) reported that acute EFT reduced delay discounting in adults with prediabetes, overweight or obesity, and a history of hypertension and/or hyperlipidemia, but did not alter demand on a food purchase task or reduce food consumed in an *ad libitum* eating task. Similar null effects of EFT on food demand in adults at risk for diabetes were reported by Stein et al. (15), despite significant reductions in delay discounting. Additionally, in two experiments, Naudé et al. (39) did not observe a significant reduction in delay discounting (Experiments 1 and 2) and cigarette demand (Experiment 2) following acute engagement in EFT in cigarette smokers, although an interaction between EFT and graphic warning labels on delay discounting was observed in Experiment 1 (lower delay discounting when exposed to EFT and graphic warning labels). Similarly, following daily exposure for 1 week to EFT or a control condition combined with either education on the Dietary Approaches to Stop Hypertension (DASH) diet or a food safety control, Hollis-Hansen et al. (23) observed greater DASH diet adherence as measured by 24 h food recalls for EFT participants, but did not observe significant decreases in sodium, fat, carbohydrates, protein, sugar, or calories purchased by mothers while grocery shopping. Mansouri et al. (40) found that daily exposure to EFT cues for 1 week did not decrease delay discounting, calories consumed in an *ad libitum* eating task, or relative reinforcing value of high-energy-dense snack foods. Finally, in a 24-week randomized controlled trial examining the effects of periodic EFT engagement combined with a weight loss intervention for overweight adults with prediabetes, participants exposed to regular engagement and instruction in using EFT did not lose more weight or improve HbA1c relative to controls, despite significant differences in delay discounting (29). For each of these examples, future research is needed to assess if these findings may be the result of procedural variations (i.e., EFT cue generation methods, cue quality, implementation or exposure to EFT cues, and different measurement methods of dependent variables) or nuances highlighting the specific conditions or populations in which interventions designed to evoke EFT are efficacious. To facilitate this work, health behavior researchers would benefit from a detailed and standardized description of methods for implementing EFT across a variety of situations.

Despite a growing body of literature describing the qualities of EFT cues that are associated with increased efficacy in the reduction of delay discounting [e.g., vividness, future-oriented, personal relevance, and positive valence; see (41–43)], the methods for generating episodic content, implementing EFT, and examining the effects of EFT on dependent measures are variable across studies and experimenters. For example, participants may generate cues independently [e.g., (27)], or with the help of an experimenter [e.g., (25)]. Participants may generate cues only once, or multiple times over the course of long-term studies. Participants may be instructed to write cues that are focused on achieving health goals, or to describe events that are pleasant and plausible. In many cases, the decisions on how EFT cues are generated and implemented are informed by the overall study design, dependent variables, research setting, and the population of interest. However, a comprehensive discussion of the relevant considerations to inform such decisions is not yet available in the published literature. Moreover, few resources are available that describe specific protocols for generating EFT cues. The details included in these protocols will be critical in future attempts at replication and application of EFT to novel contexts, as a lack of methodological transparency could contribute to an increased likelihood of type I and II errors and inhibit systematic investigation of EFT parameters that produce optimal therapeutic benefits.

The purpose of this review is three-fold: (1) to describe common methods regarding the process of EFT cue generation and implementation; (2) to provide recommendations toward current best practices for the generation and implementation of EFT cues, including control group manipulations; and (3) to discuss gaps in knowledge in the currently established methodology used to implement EFT for behavior change. The narrative review is divided into six sections: (1) the introduction; (2) cue generation methods; (3) cue content; (4) control conditions; (5) cue delivery; and (6) summary and conclusions. Each section will conclude with a summary of current best practices and future research directions.

## Cue generation methods

Two methods have been commonly used to generate EFT cues for use in studies on delay discounting and other health behaviors: interview-guided and survey-guided. Some aspects of cue generation are shared between each method. For example, depending on the study sample and research question, researchers may instruct the participants to choose events that specifically do not involve engaging in the health behavior of interest [e.g., events in which the participant is smoking cigarettes; (10)], explicitly request participants to create cues that are related to health goals (44), or instruct participants to generate a series of related cues that form a larger, connected narrative over time (45).

Below, we consider the specific procedures used in both the interview and survey-guided methods. For clarity, we refer to the single phrase or sentence beginning the event description as *tags*, and the full narrative text, including the details of the event, as *cues* (46).

### Interview-guided

During interview-guided cue generation, a researcher asks participants to identify, imagine, and describe positive but realistic events that could occur in the future (e.g., 3 months from now, 1 year from now). To form the cue, the interviewer elicits general details of the future event and asks the participant to construct an appropriate introductory sentence (i.e., a tag), such as “In 3 months, I am at the beach with family” or “In 1 year, I am exploring a new city with friends.” To ensure the personal episodic nature of these future events, researchers then lead the participant through a series of questions designed to gather more specific, personal details to create the full cue (e.g., “What will you be doing?”, “Who are you with?”, “How will you be feeling?”, “What will you be hearing and seeing?”), allowing the event to be described as if it were currently happening (21, 28, 39). The researcher may provide examples of good cues (i.e., many details and positive valence) and bad cues (i.e., few details and negative valence), and the events described in the cue should have a discrete beginning or ending that lasts <1 day.

To enhance the salience of the future event and to ensure that participants are attending to the details in the instructions, prior studies from our group have asked participants to first select a hypothetical date for the event on a digital calendar (47) before completing an event tag (e.g., “In 5 years, I am attending my child's graduation ceremony”). Participants then answer questions about valence (excitement, enjoyment, importance, and vividness) regarding the event. Afterwards, the interviewer probes for further episodic details to complete the cue and reviews a checklist of requirements [e.g., did the participant use the correct format, choose a vivid event they were looking forward to, and describe the event in the present tense; (21, 28, 30, 46)].

Cue generation methods are designed to yield cues that are at least 2 sentences in length, but may be longer depending on the level of detail. To minimize researcher bias, the interviewer should record all participant responses verbatim and only prompt participants for additional details using standardized questions. For example, if participants provide only general or terse responses, researchers can remind participants to describe the event as if they were experiencing it first-hand in the present moment. In some cases, participants may be asked to generate one or two words to remember the cue [e.g., “birthday party,” “graduation;” (10, 32)]. A full cue might read as, “In 3 years, I am attending my family reunion. We are at the pavilion in the park on a beautiful sunny day. I am excited to see my cousins and eat some of my grandma's famous potato salad.” By standardizing the EFT cue generation process, variations in cue quality (i.e., the extent to which the cue adheres to instructions) can be attributed to individual differences between participants; future research should address the possibility of cue quality moderating the effectiveness of EFT.

### Survey-guided

During survey-guided cue generation, participants independently type their answers to the questions mentioned above, typically using survey platforms such as Qualtrics or SurveyMonkey. In most studies, the survey platform emulates the procedures described in the interview-guided format, but without the need for in-person or virtual interaction with the researcher. This increases the flexibility and feasibility of the intervention, allowing it to be delivered online and to larger participant samples. A template self-guided cue generation survey (created using Qualtrics) can be seen in the Supplementary materials. The language in this template is identical to that used in several prior studies [e.g., (28, 29, 31)], although it features only a single example time frame for the EFT and ERT conditions. This example may be iterated across a range of other time frames, as is typical in prior research (e.g., 1 month−10 years; (29); see Section Time frames of future events for further discussion). Note that the language in this survey has also been used to direct an experimenter-guided interview in other studies [e.g., (47)].

### Other methods

Additionally, a robust body of literature exploring episodic thinking, both past and future, exists within cognitive psychology. In some cases, cognitive psychology researchers are interested in what mechanisms allow for EFT, including the effects of neurological and pathological conditions on the ability to engage in prospection (48). To this end, multiple standardized methods to prompt EFT have been developed, such as the Modified Autobiographical Memory Test (49), the Sentence Completion for Events in the Future Test (50), and the future-oriented Autobiographical Interview (51). To our knowledge, there have been no comparisons in studies on delay discounting and health behavior of different methods of cue generation on cue quality or behavioral change. Future methodological studies that provide these comparisons may enhance the feasibility and efficacy of EFT interventions.

### Cue generation: Current best practices and future research directions

Although both the interview- and self-guided EFT methods have been used successfully to reduce delay discounting and health behavior outcomes [e.g., (15, 52)], no studies to date have compared these methods on measures of cue quality or their efficacy for changing behavioral outcomes. Such investigations are warranted and may help identify methods for optimal implementation of EFT interventions. However, in the absence of evidence indicating differential efficacy of these methods, researchers may wish to choose a cue generation method that fits the study sample and research questions. For example, when delivering EFT in an acute, online study, it may not be feasible to utilize interview-guided cue generation methods. For trials in which EFT will be used repeatedly, interview-guided cue generation methods will increase the costs of the intervention due to increased personnel time; researchers should consider how frequently (if at all) EFT or control cues will be regenerated, and how this will impact feasibility and disseminability. Finally, some populations, such as those with limited education or literacy, may struggle with self-guided EFT cue generation. Regardless of the specific method used in cue generation, researchers may find that some participants provide terse event descriptions with minimal episodic details (e.g., “In 3 months, I am at the beach playing in the sand”). In such cases, participants could be prompted in real time (if using the interview-guided format) or, in some cases, following cue generation (if using the survey-guided format), to include more details such that the cue imagery is more vivid and engaging. However, caution is warranted in these cases, as such remediation may prevent researchers from identifying participant characteristics [e.g., depressive symptoms, working memory deficits; (53, 54)] associated with difficulties in generating cues and engaging in EFT. Identifying standardized, a priori strategies to improve EFT cues in populations that may struggle to generate quality EFT cues is an important next step in the development of EFT as a clinical tool.

## Cue content

Previous research has identified characteristics of EFT cues that influence their efficacy for changing delay discounting. Rösch et al. (41) conducted a meta-analysis to examine the effect of EFT on delay discounting, finding a medium sized effect after incorporating 174 effects from 48 articles. In sub analyses, the authors explored the effects of “core components”, finding several moderators of the effectiveness of EFT on delay discounting: cue vividness, positive valence, and content-specificity (i.e., when EFT content was related to personal goals or potential rewards in the discounting task). Additionally, future orientation and episodicity (definitional characteristics of EFT) were also indicated as important, although they were not significant moderators when compared to a group including all other control conditions. These results suggest that in addition to being future-oriented and episodic (i.e., first-person accounts of specific events), cues should be vivid and personally relevant (55). However, some aspects of EFT cues that may increase efficacy have garnered conflicting or insufficient evidence, such as positive emotional valence and goal-orientation. The following section will discuss current research on each of these characteristics in turn.

### Future orientation

Prior studies have demonstrated that future orientation of episodic thinking is necessary to produce effects on delay discounting. Thus, the events described in cues should be set in the future. Lin and Epstein (32) used a 2 × 2 between-subjects design to examine the effects of time perspective (episodic present thinking vs. EFT) and emotional valence (neutral vs. positive) on delay discounting. EFT led to significantly greater reductions in delay discounting compared to episodic present thinking; neutral and positive EFT did not differ significantly. Dassen et al. (56) used the same design to examine the effects of temporal orientation (EFT vs. episodic past thinking) and cue content (general vs. food-related) on delay discounting and caloric intake in 94 female undergraduates. Similarly, participants who engaged in EFT had reduced rates of discounting than participants who engaged in episodic past thinking; additionally, participants who engaged in food-related EFT ate fewer calories than participants who engaged in food-related episodic past thinking. Finally, Daniel et al. (57) tested the effects of EFT and episodic past thinking on hypothetical past and future discounting, finding that EFT reduced the discounting of future rewards, while episodic past thinking reduced the discounting of past rewards.

Further highlighting the importance of future orientation, episodic recent thinking (ERT), a commonly used control procedure for EFT [e.g., (12)], further highlights the importance of the future orientation of cues. ERT is an instance of episodic past thinking that involves asking participants to generate descriptions of events that have occurred recently, typically within the past day (21) or several days [e.g., “About 24 h ago, I was having lunch with an old friend. I was enjoying catching up over a bowl of soup while sitting on the patio. I felt happy to see them again”; (27, 28, 47)]. By controlling for personal relevance, vividness, and the episodic quality of cues, researchers are able to study the effect of the future orientation of cues in isolation. ERT will be covered in more detail in Section Episodic recent thinking. At present, however, the specific temporal distance that distinguishes ERT and episodic past thinking is not defined, and more work should investigate whether these conditions produce comparable estimates of decision-making.

### Time frames of future events

A potentially important consideration when utilizing EFT is choosing the time frames of the future events. Although prior work clearly indicates that future orientation is critical (see Section Future orientation), no research has systematically examined a possible moderating influence of temporal distance in EFT cues. Prior research has utilized a wide range of time frames in EFT, ranging from events within the next 6 months [e.g., (57)] or a year [e.g., (28)], to events within the next 10 years (29) or 25 years (47). Whether nearer or more distant EFT time frames differentially influence delay discounting or other behavioral measures remains unclear and should be addressed in future research.

These investigations on measures of delay discounting may be complicated by standard methods in prior research in which the time frame of the EFT cue matches the active delay in the discounting task (e.g., imagining an event in 6 months when answering, “Would you rather receive $50 now or $100 in 6 months?”). At least one prior study suggests this matching is not necessary, as O'Donnell et al. (58) reported that temporally matched and unmatched EFT cues produced comparable effects on delay discounting compared to ERT control. Nonetheless, the influence of any specific time frame or range of time frames in this and other studies could not be determined. In addition to investigating whether certain time frames are more efficacious than others, future research should also examine whether participant characteristics (if any) moderate these estimates. For example, the effects of a given range of time frames may be moderated by individual or population levels of baseline delay discounting or capacity for episodic prospection. Additionally, whether time frames that lead to greater reductions in delay discounting would translate to greater changes in health-related behaviors, such as drug use, food choices, or physical activity, remains unclear. More work is needed to understand when and for whom time frames are most efficacious.

### Vividness

The vividness of cues, as measured by counting details provided by participants or asking participants to self-report vividness, has been shown to be related to greater reductions in delay discounting in both adults and adolescents (55, 59). Thus, cues should contain sufficient details to guide participants to vividly imagine the future event.

Peters and Büchel (55) measured vividness of EFT cues by calculating an “imagery score”. While completing delay discounting tasks during an fMRI scan, participants were shown brief descriptions of previously generated EFT cues (e.g., “vacation paris,” “birthday john”). Following the task and scanning, participants rated how frequently the descriptions caused episodic prospection (1, never; to 6, always) and how vividly they experienced the prospection (1, not at all vivid; 6, highly vivid). The imagery score was the average rating of both frequency and vividness. Higher imagery scores were associated with greater within-subject differences between uncued (i.e., control) and cued delay discounting, suggesting that cue vividness may be related to the magnitude of the effect of EFT.

Using alternative methods to quantify vividness, Bromberg et al. (59) reported similar findings in adolescents. Specifically, in a structured interview, participants were prompted to provide details of an event related to specific domains of life (e.g., family, school, and recreational activities) that could occur within a future time period (e.g., 6 months and 1 year). After the prompt, participants were given 3 min to independently generate details related to the event. After the participant finished generating cues, or 3 min passed, the experimenter asked a standardized follow-up question (e.g., “Could you tell me more about where and when the event will take place, who is with you, how you feel and what you think?”). Researchers then scored audio recordings of EFT cues for internal (i.e., episodic, relating directly to the individual's possible experience of the event) and external (i.e., semantic and factual information relating generally to the event) details, reporting that more internal EFT details were associated with lower discounting rate (external details were observed too infrequently to be included in analyses), suggesting that highly detailed EFT cues may lead to greater reductions in delay discounting.

Using a similar scoring methodology, Palombo et al. (60) compared the effects of EFT on delay discounting between individuals with amnesia following medial temporal lobe (MTL) damage and healthy controls. Participants with MTL damage generated significantly fewer internal details than healthy controls; additionally, researchers observed a significant correlation between the number of internal EFT details and reductions in delay discounting (total reward obtained as a proportion of maximum possible reward) among healthy controls, but not among individuals with MTL damage. While the primary implications of this work indicate the MTL as a vital component of EFT, the relationship between details and change in reward index scores in the control group provides further evidence that vividness of EFT is a critical determinant of EFT's effects on delay discounting.

In contrast, Snider et al. (21) did not observe similar results regarding self-reported cue vividness and the effectiveness of EFT among individuals with alcohol use disorder. To measure valence of EFT and control (ERT) cues, participants rated cues on a 1–5 scale for enjoyment, importance, excitement, and vividness (as described above). Researchers compared the effects of EFT and ERT on delay discounting, including valence scores as covariates. While group assignment significantly accounted for the variance in delay discounting, none of the valence scores did (though the authors note that vividness was the only valence score that approached significance). Additionally, no differences in cue vividness were observed between the EFT and ERT groups, though this may be a result of ERT participants being asked to create cues based on events that occurred within the past day and can be considered a strength of the control condition. Additionally, ratings of vividness were self-reported by participants, in contrast to previous studies in which vividness was rated by experimenters or distinguished between episodic vs. semantic vividness [e.g., (59, 60)].

### Positive valence

Compared to negative valence, positive valence tends to increase the number of episodic details in EFT cues (61). However, the impact of the emotional valence of EFT cues on delay discounting is not fully understood. Several studies have manipulated emotional valence of future thinking or control conditions in order to understand the role this aspect of prospection plays on delay discounting, with mixed findings.

As mentioned previously, Lin and Epstein (32) used a 2 × 2 between subjects design to examine the effects of cue valence (positive vs. neutral) and time orientation (EFT vs. episodic present thinking) on delay discounting. Participants generated neutral cues (events participants were neither looking forward to nor seeking to avoid) or positive cues (events participants were looking forward to) set in the future (throughout the next 6 months) or in the present (over the next day). Participants generated a total of 12 cues (three at four possible time points); after rating cues for valence and vividness, participants generated short word cues (i.e., tags) for the four events they rated highest in vividness, which were read out loud before making choices on the delay discounting task. Compared to episodic present thinking, EFT participants had significantly lower rates of delay discounting; there were no observable differences between neutral and positive cues, or an interaction between cue valence and time orientation.

Extending the range of valence to include negativity, Bulley et al. (62) examined the effects of EFT cue valence (positive vs. negative) on delay discounting and risk-taking in undergraduates; a neutral, non-temporal cue group was included as a control. Participants first completed a delay discounting task, then viewed 10 positive, negative, or control (neutral and non-temporal) short events. The short events were created to be easily and vividly imaginable, and plausible for students. After ranking the events for relevance, the five highest ranked events were used as cues during the delay discounting and risk-taking tasks. Before each choice in the delay discounting task, participants were instructed to imagine themselves personally experiencing the positive or negative future events in as much detail as possible; future events timings were roughly matched with the receipt of delayed rewards in the delay discounting task. Participants were not instructed to imagine the neutral, non-temporal events as occurring in the future, but as they typically occur. Results indicated that participants in the negative and positive future thinking groups discounted the value of future rewards less steeply than individuals in the control (neutral and non-temporal) group. No significant differences in discounting rates were observed between the positive and negative emotional valence groups.

In a similar experiment, Calluso et al. (63) examined the effects of cue valence (i.e., positive, negative, and neutral) on delay discounting. Participants first completed a baseline delay discounting task; afterwards, participants completed a questionnaire to generate future thinking cues. Participants listed vivid personal events that could potentially occur at each delayed reward time period and listed events at each level of cue valence; cues were rated on personal relevance, arousal, and valence. Three days later, participants returned to the lab to complete three cued discounting tasks (one for each level of valence) in counterbalanced order. In these tasks, the future event was described as occurring at the same time as when the larger later reward would be delivered (e.g., “10 euros now or 60 euros at the end of the semester [90 days]?”) Participants were not explicitly instructed to imagine the future events while making their choice, but to consider the future event in order to learn when the delayed reward option would be delivered. Results revealed decreased rates of discounting in all valence conditions compared to the baseline. Indeed, discounting rates were significantly different between all valence conditions, with the highest rates occurring in the baseline, followed by the negative, neutral, and positive conditions.

In contrast, Liu et al. (64) reported that the effects of EFT varied as a function of emotional valence in a series of experiments examining the effect of positive, negative, and neutral prospection on delay discounting in undergraduate students at a Chinese university. In each experiment, participants completed a baseline measure of uncued delay discounting followed by a delay discounting task including an emotional valence manipulation (Experiment 1: positive events; Experiment 2: negative events; Experiment 3: neutral events). To manipulate valence in each experiment, participants were shown 10 future events that could reasonably be experienced by a college student. Positive events were occurrences that participants would be likely to look forward to (e.g., classmate party); negative events were occurrences that participants would be likely to avoid (e.g., failed exam); neutral events were repetitive tasks that were considered emotionally neutral (e.g., doing laundry). Participants imagined each event and rated events on valence and relevance; the five events highest in valence and personal relevance were then presented as short, two- to three-word phrases directly underneath the larger later options in the delay discounting task. No explicit temporal delays were included in the future event phrases, although participants were told to imagine the events occurring on the day the delayed reward would be delivered (as in the study by (63)). The choice options and event phrase were only presented for 4 s. Across three experiments, these versions of positive, neutral, and negative EFT significantly increased, had no effect, and significantly decreased preference for the larger later reward, respectively, compared to a baseline condition.

In a replication of Liu et al. (64), Zhang et al. (65) improved upon some of the methodological shortcomings of the previous study. As in Liu et al. (64), researchers first asked participants to complete a baseline measure of delay discounting, followed by a delay discounting task including a short description of a positive, negative, or neutral event. Events were defined and identified in a similar manner as in the original study; the presentation of the future event and choices during the delay discounting task were nearly identical, with the important difference that participants in Zhang et al. (65) were able to view the choice options and the event phrase until they made a response indicating that they were ready to select an option; allowing participants to view the cue for longer than 4 s may have enabled more vivid prospection. Additionally, participants completed a third delay discounting task in which the event phrases were again presented underneath the larger later rewards; however, in this condition (i.e., the no-prospection condition), participants were not instructed to imagine the future events occurring on the day of the delayed reward delivery (in contrast to the prospection condition). Participants experienced the prospection and no-prospection condition trials equally and in pseudorandom order; to help participants distinguish between conditions, the words “imagine” or “no” were presented before the trial choices were displayed, signifying the prospection and no-prospection conditions, respectively. After completing the delay discounting task in each condition, participants rated how often each episodic tag evoked prospection and the vividness of prospection on scales from 1 to 7. Results suggest that participants in the prospection condition reported engaging in prospection significantly more frequently than during the no-prospection condition; additionally, no differences in vividness were observed between valence groups. Results revealed that emotional valence significantly interacted with prospection, with *post-hoc* comparisons revealing again that positive EFT decreased delay discounting and negative EFT increased delay discounting, replicating the findings of Liu et al. (64).

In summary, Bulley et al. (62) and Calluso et al. (63) observed reductions in delay discounting compared to baseline or control conditions even using negative valence EFT cues. Lin and Epstein (32) observed reductions in delay discounting between the neutral and positive EFT conditions; similarly, Calluso et al. (63) observed decreased discounting rates after exposure to neutral cues compared to baseline. It is unclear why Liu et al. (64) and Zhang et al. (65) both observed conflicting results regarding the effects of negative and neutral valence cues on delay discounting (i.e., increased rates of delay discounting).

Interpretation of these discrepant results is challenging due to methodological differences between the studies regarding EFT generation and implementation, control groups, and measures of delay discounting. Despite these challenges, some speculation is warranted. The negative EFT intervention utilized in Liu et al. (64) and Zhang et al. (65) may have induced stress. In prior studies, stress exposure is cross-sectionally, longitudinally, and experimentally associated with elevated discounting [for meta-analysis, see (66)]. Likewise, in more recent studies, narrative simulations of stress in the form of negative income shock and other personal disruptions have been shown to increase delay discounting [e.g., (28, 67)]. Thus, the increases in delay discounting observed by Liu et al. (64) and Zhang et al. (65) following negative EFT may be mediated by stress. However, why similar effects would not have also been observed by Bulley et al. (62) and Calluso et al. (63) in their negative EFT manipulations remains unclear. At present, more research is needed to clarify the role of positive valence on the effectiveness of EFT.

### Personal goal orientation

Significant work within cognitive psychology suggests that integrating personal goals into future thinking cues is an important aspect of effectively imagining future events [see (68)]. Indeed, EFT cues describing personal goals leads to greater activation of brain regions that contribute to future thinking, such as the medial prefrontal cortex and posterior cingulate cortex, compared to EFT cues that describe non-personal goals (69). Broadly speaking, research has focused on financial-goal oriented EFT and health-goal oriented EFT.

O'Donnell et al. (70) examined the effects of financial goals in EFT on delay discounting; 104 participants (non-smokers between 19 and 35 years old) were randomly assigned to one of four conditions in a 2 × 2 design (EFT/ERT, goal/general). Goal-oriented cues were events related to future financial goals for EFT participants (e.g., “In 1 month I am purchasing a new road cycle and helmet at the local shop with my friend. I am feeling excited to have a new way to get around town and am proud of myself for saving up for a high quality model”) or recent positive events related to spending for ERT participants (e.g., “24 h ago I purchased some headphones online. I was at home on my computer. I felt excited to have new headphones to wear while exercising or just relaxing”). General (no goal orientation) cues were typical EFT or ERT cues describing events participants were looking forward to or enjoyed recently. Participants generated episodic details for each event (e.g., where they were, how they felt, whom they were with, what they did), which were then rated for salience, valence, arousal, and vividness. In total, participants completed seven cues each. Participants then completed a delay discounting task in which participants read their cues aloud before beginning a block; the cues were printed and visible to participants as they made their choices in each block. For EFT participants, cues were presented such that the timing of the future events matched the delivery of the larger later rewards; for ERT participants, cues were randomly presented with delay blocks. Results revealed that both EFT groups (goal-oriented and general) demonstrated less delay discounting than both ERT groups; additionally, the participants who generated EFT cues including financial goals demonstrated less delay discounting than the participants who generated general EFT cues. There were no observable differences between the goal-oriented and general ERT control groups.

O'Neill et al. (22) demonstrated that health-goal EFT cue exposure can lead to reduced caloric intake in women with overweight in naturalistic settings. Participants created EFT cues that were explicitly paired with future health goals; participants first generated future health goals, followed by future events that could occur in 3 weeks. Participants were asked to pair their health goals with the future events to create goal-oriented EFT cues. O'Neill et al. (22) provided the following example of a health-goal oriented EFT cue: “In 3 weeks, I will go to the concert with my friend. We will sit near the back so we can chat more easily. I will be feeling strong and proud of myself after having achieved my goal of going to the gym three times per week. I will be feeling excited and happy to see the band play for the first time.” ERT control participants described regular habits they enjoyed and paired them with events that occurred in the past day. Participants exposed to EFT cues consumed fewer calories in an ad-libitum eating task. While this experimental design suggests that health-goal oriented EFT is effective for this behavioral outcome, this study did not explicitly arrange comparisons of the relative efficacy of health-goal oriented and general EFT.

In a subsequent study, Hollis-Hansen et al. (14) observed that health-goal, process-oriented EFT cues (71) led to reduced calories purchased during an online grocery shopping task compared to a money-savings control group. Health-goal, process-oriented cues combine future goals related to health with concrete behaviors or actions that may increase the likelihood of meeting the goal (e.g., “In 1 month, I am shopping for groceries. I am putting vegetables and lean proteins into my cart. I am feeling proud of myself for making a choice that will make it easier for me to eat healthier.”). Hollis-Hansen et al. (14) included a second experiment comparing health-goal, process-oriented EFT cues with both general EFT cues and general ERT cues. Both EFT groups purchased significantly fewer calories compared to the ERT control, but there were no significant differences in calories purchased between the two EFT conditions.

Finally, Athamneh et al. (44) extended this line of work by examining the effects of health goals in EFT on both delay discounting and other behavioral measures (i.e., food and cigarette demand and craving) in clinically relevant samples (i.e., smokers and individuals with obesity). In Experiment 1, the researchers first examined the effects of health-goal EFT, general EFT, and general ERT on delay discounting, cigarette demand, and cigarette craving in current smokers. Health-goal EFT participants were instructed to “associate their events with any health goal that they were looking forward to” [(44), p. 3]; goals did not have to be explicitly related to smoking cessation, but could be. Health-goal EFT and general EFT led to reduced delay discounting compared to ERT, but no differences in this measure between health-goal EFT and general EFT were observed. Interestingly, health-goal EFT exhibited a significantly lower intensity of demand for cigarettes (i.e., self-reported consumption when cigarettes were free) than both the general EFT and general ERT groups. Additionally, health-goal EFT participants exhibited significantly higher elasticity of demand (i.e., a greater reduction in consumption as a function of increased price) and lower craving for cigarettes compared to ERT participants, while the general EFT group was not significantly different than the ERT group. In Experiment 2, the researchers compared the effects of health-goal EFT and general EFT to health-goal ERT and general ERT on delay discounting, demand for fast food, and craving for fast food in individuals with obesity. To generate health-goal ERT cues, participants described recent events that were associated with health-related activities (e.g., weight loss and physical activity). Replicating the results of the first study, no differences in delay discounting were observed between health-goal and general EFT, although both EFT groups had significantly less discounting than both ERT groups. No differences in delay discounting were observed between health-goal oriented ERT and general ERT. On measures of craving, intensity of demand, and elasticity of demand, only health-goal EFT participants exhibited significantly different values than any other group (i.e., lower craving and intensity and higher elasticity). The effect of health-goal EFT cues were most evident on intensity of demand, where health-goal EFT participants had significantly lower intensity than all other groups.

These observations support cognitive and neurological data suggesting that personally-oriented goals are important aspects of prospection; however, the impact of goal-oriented EFT cues on delay discounting may be dependent upon the nature of the goals described and participant characteristics. Notably, in both studies reviewed above, goal-oriented EFT was only effective beyond general EFT when the content of the goals (e.g., financial or health-goals) was congruent with the behavioral task in which the goals were presented (i.e., delay discounting tasks may appear as a financial decision making task, demand and craving for fast food or cigarettes may appear as a health decision making task). More work is needed to clarify the role of goal orientation in EFT cues and their impact on delay discounting and other relevant health behaviors, particularly when goals are incongruent or unrelated to dependent variables.

### Cue content: Current best practices and future research directions

To be most efficacious for behavior change, EFT cues should be vivid (see Section Time frames of future events) and, moreover, incorporate personally relevant goals that are related to the behavioral outcome of interest (see Section Positive valence). Additionally, positive valence in EFT cues may be more efficacious than negative cues in reducing delay discounting, although the conditions that moderate this effect are not clear (see Section Vividness). Future research should seek to clarify the role cue valence and personal goal orientation play in the efficacy of EFT, and better understand the impact of different time frames.

If EFT technologies are to be successfully translated for use by community health providers, it is imperative that providers are able to implement EFT interventions with fidelity (72). This highlights the need for an easy to implement and objective means to measure EFT cue quality. While the Autobiographical Interview, originally developed to identify episodic and semantic details of autobiographical memory (51), has been adopted by cognitive neuroscience researchers to quantify and score internal (episodic) and external (semantic) details in future thinking cues (73), this method has not been used to examine the effects of cue quality on delay discounting or other behavioral outcomes. Future research should seek to develop methodologies to measure cue quality to enable further research with the goal of refinement of EFT technologies for behavior change. Additionally, creating standardized methods for improving the quality of participant-generated EFT cues to be used by community health providers would increase the utility of EFT as a clinical tool.

## Control conditions

There are a wide variety of control procedures that have been used in the EFT literature, from no active control manipulations [i.e., uncued assessment; (55)] to control conditions designed for use in clinical settings (46). The purpose of control conditions in EFT studies is to provide an experimental condition in which participants are exposed to some aspects of the independent variable (EFT) which may be responsible for an unknown magnitude of changes in the dependent variable. This allows for an examination of the effect of a single aspect of the independent variable in the active (EFT) condition. In the sections below, we provide an overview of each of the control methods that have been used in studies of EFT and delay discounting while highlighting relevant considerations for each method (see (74), for further discussion of EFT control methods).

### Episodic recent thinking

As mentioned previously (Section Future orientation), one widely used control procedure is ERT, where participants vividly describe events that have occurred in the recent past (10, 12, 18, 21, 28, 32). ERT controls for the personal, vivid, and episodic nature of imagined events, as well as time and effort involved in cue generation, therefore isolating the effects of prospection in EFT by shifting temporal orientation to the recent past. The time frames used in ERT have varied considerably, ranging from a few hours from the present to within the past 12 days (21, 27, 70). It is possible that vividly imagining events that have occurred too far in the past may inadvertently cause an individual to consider future possibilities (74). This may occur due to the process of imagining future events, in which memories of past experiences are theorized to enable an individual to engage in prospection of future events [see constructive episodic simulation hypothesis; (75)]. Daniel et al. (57) demonstrated that vividly imagining retrospective events reduced delay discounting compared to a recent and near-future control (i.e., within 4 h before and after present), with the effect of retrospective events on delay discounting increasing as the events were further from the present (i.e., from 1 day ago to 6 months ago). Additionally, Lempert et al. (76) observed that imagining positive autobiographical memories reduced delay discounting compared to a no-memory retrieval control. It is unclear as to how far back in time retrospection must be removed from the present before it is likely to cause prospection.

### Standardized episodic thinking

Considering the wide variety of control procedures and the possibility for ERT to cause prospection, Hollis-Hansen et al. (74) designed an experiment to test a novel control procedure: standardized episodic thinking (SET). SET utilizes the same methods of generating EFT and ERT cues, but standardizes participants' recent experiences and the time frames in which participants describe recent events. To test the effect of the SET condition on delay discounting, 53 participants were randomized to EFT, ERT, or SET groups. All participants chose three preferred mobile (i.e., cell phone) games to play for 5 min each before generating cues. EFT and ERT participants generated three cues by describing an event, rating the event for vividness and liking, then describing the event in vivid detail. SET participants generated three cues about the mobile games played previously. Hollis-Hansen et al. (74) describe an example of an SET cue:

About 5 minutes ago I was playing Bubble Witch in a beige study room at [the University]. I was playing as a witch and guiding her wand toward similarly colored bubbles in order to pop them. I was releasing the owls as I popped the bubbles. I was feeling excited as I was making my way to the top. (p. 7)

Participants in each group then completed a delay discounting task in which they first read their cues and were instructed to remember their cues as they made their choices. Participants also rated how frequently and vividly they recalled their cues as they completed the delay discounting task; importantly, there were no differences in how vividly participants from each group imagined their cues during the task. EFT participants discounted future rewards less steeply than ERT and SET groups; no differences between ERT and SET groups were observed. The results suggest that SET is a reasonable control condition that prevents participants from inadvertently engaging in prospection as a result of retrospection. However, SET as a control condition has some limitations. The recent activity used to standardize participants' recent experiences (i.e., video games) may not be preferred by all study populations. Additionally, in the present study, participants in each group generated three cues; thus, participants played three mobile games for 5 min each. Experiments in which participants generate more cues (e.g., six or seven) would require twice as much time playing the games before cue generation. This could be offset by reducing the allotted gaming time (e.g., 2.5 min for each game), but it is unknown how this would affect the vividness of the cues generated by participants.

### Health information thinking

Another recently developed control condition is the health information thinking (HIT) condition, pioneered by Sze et al. (24) and further developed by Rung and Epstein (46) for use in experiments in which EFT is implemented multiple times per day or for extended periods of time (i.e., clinical settings). Rung and Epstein (46) identified some shortcomings of ERT as a control condition for clinical applications. In order to keep ERT cues “up to date”, cues with recent temporal proximity (i.e., 12 h and 1 day) would have to be regenerated prohibitively often. This frequency of regeneration would need to be matched in the EFT group, which could be challenging for participants to generate novel, detailed future experiences. Additionally, the concern of ERT inadvertently causing prospective thought may increase as participants engage in ERT regularly. Finally, ERT as a clinical control fails to hold constant participants' expectation of improvement or perceived helpfulness between ERT and EFT groups. HIT was developed as a control specifically to address these concerns.

To test HIT, Rung and Epstein (46) recruited 254 participants using Amazon Mechanical Turk. Participants were randomly assigned to EFT, ERT, or the novel HIT condition; EFT and ERT participants generated cues by identifying an event at the relevant time point, rating the event for vividness, likability, importance, and excitement, then describing the events in detail. HIT participants read six paragraphs, each discussing a different health topic; topics included prediabetes, electronic cigarette use, physical activity, nutrition labeling, sleep, and depression. After reading the paragraph, HIT participants described what they had read in a single sentence. HIT then participants rated the informational paragraphs for likability of learning the information, importance of learning the information, excitement while learning the information, and usefulness of learning the information. Afterwards, HIT participants added more details to their single sentence description of the informational paragraph, addressing how the information fit into what they already know, what the paragraph made the participants think, how the information might be used, and how it made them feel. The benefit of creating cues focusing on recently learned health information is that it may create the expectation of improvement for control participants while controlling for time and effort in cue generation between groups. A complete HIT cue in response to an informational vignette on glycemic index may read: “I learned that the glycemic index (GI) of a food indicates how much change in blood glucose can be expected from eating that food. It is very important for me to maintain my blood glucose level. I think it would be very helpful for me to know more about GI to maintain my glucose level.”

Following cue generation, participants in each group completed an adjusting amount delay discounting task (77) in which the larger later delays were the same as the timeframes of the EFT cues; additionally, EFT cues were presented during the task with their matching delay block. ERT cues were presented with the most recent cues corresponding with the most temporally proximal delays in the task; HIT cues were matched with delays according to the order in which participants generated cues. For all groups, full cues were presented before beginning a delay block; during individual trials of the block, cue tags (i.e., the first sentence in which participants identify the event or the paragraph) were visible with the instruction to keep the tag in mind as participants made their choices. Following a delay block, participants indicated how vividly and frequently they imagine their cues throughout the block.

Area under the curve (AUC) values were highest in the EFT groups compared to ERT and HIT groups; no differences in AUC were observed between the ERT and HIT groups. However, exploratory equivalence analysis indicated that AUC values between ERT and HIT groups were not statistically equivalent, with the HIT group demonstrating slightly steeper rates of discounting compared to the ERT group. Compared to EFT and ERT cues, HIT cues took significantly longer to generate and were imagined less frequently during the delay discounting task; additionally, HIT cues were rated as significantly less positive and vivid than EFT cues. Controlling for these differences in secondary analyses did not result in different findings from primary analyses. Overall, HIT appears to account well for non-specific treatment factors (i.e., time spent generating cues, answering questions about cues). Importantly, HIT may engender an expectation of improvement in clinical samples similar to EFT, though this was not measured directly in the present study and would likely require future researchers to create informational paragraphs relevant to the behaviors under study. Rung and Epstein (46) identify a few key areas where more research is needed: (1) how discrepancies in vividness, positive valence, frequency, and time taken to generate HIT vs. EFT cues may affect dependent variables other than delay discounting, and (2) the effects of HIT vs. EFT in clinical populations more generally.

### Other control conditions

Other controls, such as cues based on third party narratives (11), no cue conditions (28), and semantic future thinking cues (17, 78), have also been used. Cues based on third party narratives have involved participants reading a travel blog and generating recent thinking cues based on the events in the blog. In no cue conditions, participants do not generate any cues but complete the same dependent measures as EFT participants.

Semantic future thinking (SFT) mirrors EFT, but involves only generating semantic details (i.e., external and non-personal) about future events. By controlling for future orientation, SFT allows for a comparison which isolates the effect of episodicity in the EFT condition. Chiou and Wu (17) demonstrated that EFT reduced delay discounting and cigarettes consumed compared to SFT in a sample of smokers with an intention to reduce or quit smoking; similarly, Wu et al. (78) demonstrated that EFT reduced delay discounting in undergraduates compared to SFT in two experiments. Although there are relatively few studies using SFT as a control method for EFT [see (41)], these results suggest that episodicity (i.e., imagining a personal experience) is also an important component of EFT.

### Control conditions: Current best practices and future research directions

Researchers should choose a control condition that minimizes the potential for confounding variables to threaten internal validity and which is compatible with the intervention. It is important to consider the number of exposures to cue generation, participants' expectations of improvement when deciding which control condition to utilize, and the likelihood of temporally distant past thinking to cause prospection. Future research should examine the feasibility of substituting ERT with SET during in-lab experiments, or extend the use of HIT to clinical intervention studies.

## Cue delivery

Once generated, EFT or control condition cues may be presented during decision-making tasks [e.g., (62)] or in the natural environment (e.g., (29)) to prompt episodic thinking. These cues may be presented in several forms (e.g., text and audio), at varying frequencies and durations. Below, we review the methods used in prior studies, while highlighting several considerations relevant for both laboratory studies examining acute EFT and clinical studies examining repeated EFT delivery.

### Text and audio cue delivery

Historically, EFT cues have been presented to participants using either text or audio formats, and occasionally both (10–12). Use of audio cues has typically featured recordings of the text-based cues, recorded in the participant's own voice (10, 11, 24).

Recent work has demonstrated that EFT cues may even be effective when drawn (79), although additional investigations are required. If this effect is robust in future research, drawn EFT cues may be more appropriate for children or populations with low education or literacy. Again, more work is needed to compare the efficacy of this and other formats, and to explore potential moderators of this effect (e.g., reading level).

No other studies to our knowledge have systematically compared the efficacy of cues delivered in different formats, although significant effects on behavioral measures have been observed across studies. Thus, the effects of EFT appear robust against the specific control method chosen.

### Frequency, duration, and timing of cue exposure

The majority of work examining the effects of EFT on delay discounting and other behavioral measures has consisted of a few or single exposures to cues before measuring their impact. Recently, more work has been designed to explore the feasibility of longer-term, multiple exposure to EFT cues, in both the laboratory and naturalistic settings (24, 25, 31, 52). This emerging body of research shows encouraging results for the translation of EFT technologies toward health behavior change interventions. To maximize the impact of EFT, further research should refine aspects of the implementation of EFT cues, such as the frequency with which participants are exposed to cues (e.g., twice vs. thrice per day), the duration of cue exposure (e.g., 1 week vs. 2 weeks), and the timing of cue exposure in relation to the behaviors of interest (e.g., EFT engagement before meals or grocery shopping).

### Cue delivery considerations: Current best practices and future directions

In both laboratory and clinical research, little work has investigated the effects of different cue formats, frequencies, durations, or timing on delay discounting or health behavior outcomes. More work is needed to inform empirical recommendations for best practice. Nonetheless, several research design choices that may influence EFT's efficacy and feasibility should be considered. For example, regarding cue format, text cues may be more appropriate for laboratory assessments in which participants are expected to continuously attend to a computer screen or other media; in contrast, audio cues may be more appropriate for other laboratory assessments (e.g., cigarette self-administration) in which participants are not attending to a computer screen and text cues may be less salient. Likewise, in clinical studies that implement repeated and remote delivery of EFT in the natural environment, text cues may be more appropriate because use of audio cues may limit the feasibility of the intervention if some participants feel uncomfortable listening to personalized event descriptions in the company of peers (e.g., at work).

Regarding the timing of exposure to EFT cues in clinical studies, the most appropriate times of day to prompt episodic thinking may be specific to the health behavior(s) under investigation. For example, clinical trials that target changes in dietary intake and weight loss [e.g., (24, 29)] may wish to target meal times or other times of day participants report food challenges (e.g., prior to bedtime). In contrast, for studies on alcohol or other substance use, EFT cues may be most efficacious if delivered prior to times of day that individual participants typically experience the greatest craving or most frequently engage in substance use. Additional recommendations await future research.

## Summary and conclusions

The present narrative review described current research utilizing EFT for the purpose of modulating delay discounting and other behavioral outcomes, with the goal to enable future researchers to better understand and implement EFT. Prior research methods, findings, gaps in knowledge, and considerations of best practice were reviewed related to EFT cue generation, content, control methods, and cue delivery. As significantly more work is needed in order to refine the use of EFT for behavior change, gaps in knowledge and future research directions were discussed.

**Figure 2:**
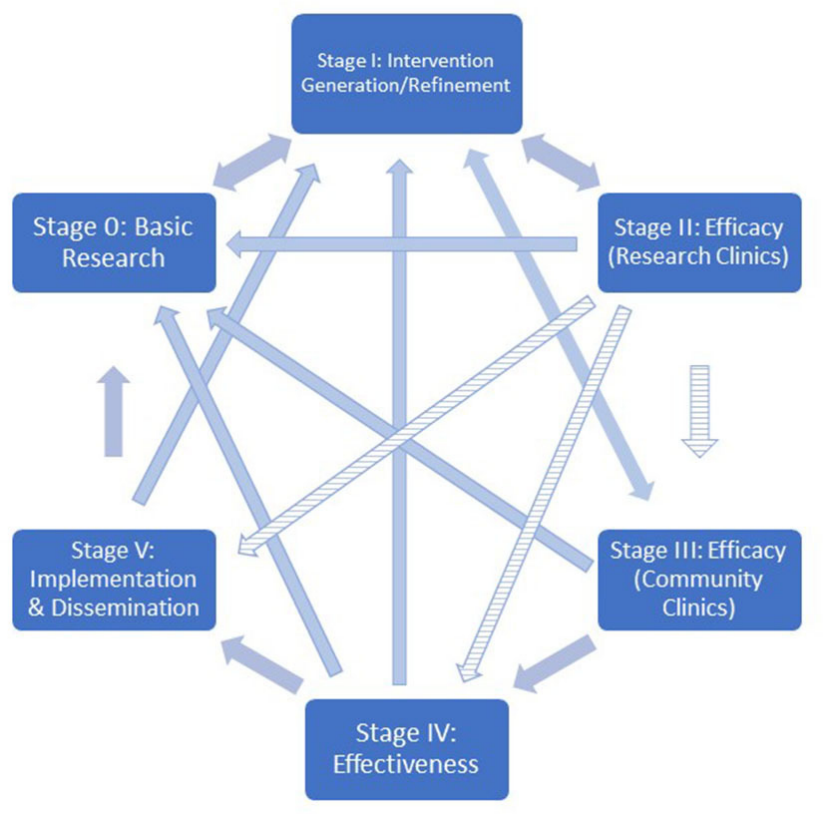
Recreation of Figure 1 from Onken et al. (72). Depiction of the NIH stage model. Notched arrows denote pathways that should be considered with caution.

Though early in development, EFT appears to be a promising intervention for a range of health behaviors. In future research, the NIH Stage Model may aid in further development of this intervention. This model describes a dynamic approach for facilitating translation of basic science into maximally feasible, effective, and generalizable clinical interventions (72). The model includes six stages, with research on the intervention's mechanisms of action as a focus in each stage (see Figure 2).

*Stage 0:* Research in this stage includes basic science findings (e.g., measurement and assessment of putative mechanisms) that contribute to intervention development.

*Stage 1:* Basic science findings are translated into novel behavioral change interventions and further refined, modified, and adapted to enable easier implementation and increased potency.

*Stage II:* Efficacy trials are conducted in which the behavior change interventions are tested and experimentally compared to controls within environments and using methods designed to maximize internal validity (i.e., in research settings, with research providers delivering the intervention).

*Stage III:* Strongly-controlled experimental efficacy trials are extended to real world community settings or implemented by community providers, while researchers carefully monitor intervention fidelity to maintain internal validity.

*Stage IV:* Effectiveness studies, in which behavioral interventions are deployed in community settings using community providers, to establish external validity of the intervention across the target population.

*Stage V:* Implementation and dissemination research, in which best practices of intervention delivery are explored.

We believe that the majority of work developing EFT as an intervention has occurred in stages 0 [e.g., (80)] and I [e.g., (11)], with some recent work into stage II (e.g., (29)). To our knowledge, no studies have crossed into stage III efficacy research or beyond; that is, EFT has not yet been examined in clinical contexts with community providers delivering the intervention.

Intervention development using the model does not necessarily progress linearly (i.e., from stages II to III); it is common for work in stages II, III, and IV to reveal the need for stage I research aimed at improving efficacy and feasibility. Indeed, this narrative review has described and identified an array of stage I research targets relevant to the development of EFT as an intervention targeting delay discounting to modulate health behavior. Research to develop the most potent form of EFT will benefit from more researchers adopting the NIH Stage Model, including a focus on the putative mechanisms causing the behavior change following EFT. We hope that the present review will spur additional stage I research in order to inform future stage II research.

In conclusion, EFT is an emerging clinical intervention that may have promising implications for a broad array of health behaviors. In the past decade, a growing body of literature has emerged that can be used to guide decisions regarding methodology and implementation; however, many questions remain unanswered. More stage I research—especially that which further refines methods, identifies for whom the intervention is efficacious, and confirms mechanisms of action—is needed to progress EFT as a feasible clinical intervention.

## Author contributions

JB and JS contributed to conception, writing, and editing. Both authors read and approved the submitted version.

## Funding

The authors' time in preparing this manuscript was partially supported by National Institutes of Health Grant R01DK129567 (JS) and the Institute for Critical Technology and Applied Science (ICTAS) Doctoral Scholar Program at Virginia Tech (JB).

## Conflict of interest

The authors declare that the research was conducted in the absence of any commercial or financial relationships that could be construed as a potential conflict of interest.

## Publisher's note

All claims expressed in this article are solely those of the authors and do not necessarily represent those of their affiliated organizations, or those of the publisher, the editors and the reviewers. Any product that may be evaluated in this article, or claim that may be made by its manufacturer, is not guaranteed or endorsed by the publisher.
