# Supplementary material for: Putting prospection into practice: Methodological considerations in the use of episodic future thinking to reduce delay discounting and maladaptive health behaviors
Source: Front Public Health. 2022 Nov 3;10:1020171. doi: 10.3389/fpubh.2022.1020171 (PMC9669959; doi:10.3389/fpubh.2022.1020171)
Supplement: Supplementary file 1 [file Table_1.DOCX]

To download the Qualtrics survey, follow this link: <https://osf.io/5w6qk/>

Download the file called “ERT-EFT cue generation template.qsf”. Import this file Qualtrics to recreate the ERT/EFT cue generation survey template.
